# Association of the *interleukin*-*12* polymorphic variants with the development of antibodies to surface antigen of hepatitis B virus in hemodialysis patients in response to vaccination or infection

**DOI:** 10.1007/s11033-013-2809-7

**Published:** 2013-10-25

**Authors:** Alicja E. Grzegorzewska, Piotr M. Wobszal, Anna Sowińska, Adrianna Mostowska, Paweł P. Jagodziński

**Affiliations:** 1 Department of Nephrology, Transplantology and Internal Diseases, Poznań University of Medical Sciences, 49 Przybyszewskiego Blvd, 60-355 Poznań, Poland; 2 Department of Computer Science and Statistics, Poznań University of Medical Sciences, 79 Dąbrowskiego Str, 60-529 Poznań, Poland; 3 Department of Biochemistry and Molecular Biology, Poznań University of Medical Sciences, 6 Święcickiego Str, 60-781 Poznań, Poland

**Keywords:** Anti-HBs, Gene polymorphism, Hemodialysis, Infection, *Interleukin-12*, Vaccination

## Abstract

Cytokines, involved in the T-helper 1 system, play a role in the regulation of hepatitis B virus (HBV) clearance and the immune response to HBV antigens during natural infection or planned vaccination. Our aim was to examine whether the polymorphic variants of *IL*-*12* are equally associated with development of antibodies to HBV surface antigen (anti-HBs) in hemodialysis (HD) patients in the case of HBV vaccination or HBV infection. The *IL*-*12A* rs568408 and *IL*-*12B* rs3212227 polymorphisms were analyzed in relation to anti-HBs development in 602 HD patients with negative antibodies to HBV core antigen (anti-HBc) who were hepatitis B vaccinated (group I) as well as in 237 anti-HBc positive HD patients who were infected with HBV in the past (group II). In group I, 199 patients did not develop an anti-HBs titre >10 IU/L (subgroup Ia), whereas in group II, 55 patients did not develop an anti-HBs titre >10 IU/L (subgroup IIa). Patients of groups I and II that developed an anti-HBs >10 IU/L were included into subgroups Ib and IIb, respectively. In hepatitis B vaccinated HD patients, development of a protective anti-HBs titre was positively associated with vintage of renal replacement therapy (RRT), chronic glomerulonephritis as a cause of RRT, and GA rs 568408 *IL*-*12A* (OR 1.6, 95 % CI 1.0–2.5, *P* = 0.035), but a frequency distribution of this genotype between responders and non-responders was not significant when the Bonferroni correction was applied. In HBV infected HD patients, anti-HBs development was positively associated with AC rs3212227 *IL*-*12B* (OR 8.0, 95 % CI 2.6–24.9, *P* < 0.001), whereas HBsAg positivity, AA rs3212227 *IL*-*12B* (OR 0.3, 95 % CI 0.1–0.7, *P* = 0.007), and CC rs3212227 *IL*-*12B* (OR 0.1, 95 % CI 0.03–0.6, *P* = 0.011) were negative predictors of positive anti-HBs phenotype. When the Bonferroni correction was applied, if appropriate, these associations remained significant. In HD patients, the studied *IL*-*12* polymorphic variants seem to be associated with the anti-HBs phenotype (a) with borderline significance for *IL*-*12A* in hepatitis B vaccinated patients, and (b) significantly for *IL*-*12B* in patients who underwent natural HBV infection.

## Introduction

Antibodies to the surface antigen of the hepatitis B virus (anti-HBs) are specific neutralizing antibodies indicative of either an immune response triggered as a result of having received a vaccine containing the surface antigen of the hepatitis B virus (HBsAg) or active immunity to the hepatitis B virus (HBV) as a result of prior infection with HBV having HBsAg in its structure. Anti-HBs in the bloodstream may also result from passive immunity created by injection of hepatitis B immunoglobulin for post-exposure prophylaxis.

According to the current recommendations, protection of hemodialysis (HD) patients against HBV infection should include hepatitis B vaccination using licensed hepatitis B vaccines given at 0, 1, 2 and 6 months in the dose of 40 μg each administered by the intramuscular route at one site. Patients who did not respond to the primary vaccine series should be revaccinated with three additional doses and retested for response [1]. The development of this advanced vaccination strategy still does not elicit the adequate anti-HBs response in 20 % HD vaccinees [2]. Therefore, a substantial number of HD patients is not adequately protected against HBV infection.

Natural HBV transmission to the human body leads to the appearance of specific seromarkers, among them HBsAg, antibodies to HBV core antigen (anti-HBc), and anti-HBs. The development of anti-HBs used to be associated with the disappearance of HBsAg. Anti-HBc are established markers of current (in IgM class) or past (in IgG class) HBV infection. If anti-HBs do not appear during the HBV infection, it results either in HBsAg carrier status, which is associated with detectable HBV deoxyribonucleic acid (DNA) in about 65 % of infected HD patients [3, 4], or in the occurrence of isolated anti-HBc positivity (anti-HBc positive individuals are both HBsAg and anti-HBs negative), which in HD patients may be associated with detectable HBV DNA [5]. HBV replication may contribute to morbidity and mortality related to HBV-associated diseases [6–8]. Ineffective hepatitis B vaccination is predictive for the prevalence and incidence of both HBsAg [9] and anti-HBc [10, 11] positivity.

Interleukin (*IL*)-*12* is a heterodimeric proinflammatory cytokine composed of a 35 kDa light chain and a 40 kDa heavy chain. *IL*-*12* plays a key role in the regulation of the immune response to HBV antigens during spontaneous infection [12–14] or planned vaccination [15, 16]. *IL*-*12* is a member of the cytokine network, which includes pro- and anti-inflammatory bioactive peptides. This network may be influenced by multiple factors, such as blood transfusions [17], stress [18], iron status [19] and many others, resulting in changes in serum levels of interleukins. The concentrations of these cytokines, among them *IL*-*12*, reflect actual somatic and behavioral status, whereas genotypes are not influenced by internal and external signals.

The light (35 kDa) and heavy (40 kDa) chains of *IL*-*12* are encoded by the *IL12A* and *IL12B* genes, respectively. The 3′untranslated regions (UTRs) influence the amount of translated protein [20], therefore the single nucleotide polymorphisms (SNPs) *IL12A* G>A (rs568408) and *IL12B* A>C (rs3212227), located in the 3′ UTR, are suspected in the modulation of *IL*-*12* levels [21]. Polymorphisms and haplotypes in *IL*-*12B* have already been directly associated with *IL*-*12* production in previous studies [22, 23]. Moreover, the number of polymorphisms located in *IL*-*12B* is limited and these SNPs display significant linkage disequilibrium [22]. Therefore, polymorphisms in the 3′ UTR region of *IL12A* (rs568408) and *IL12B* (rs3212227), influencing *IL*-*12* levels, might also affect the immune response to HBV antigens.

A recent study [24] has shown no association with HBV persistence and *IL*-*12A*, whereas the *IL*-*12B* promoter S allele was associated with non-responsiveness to HBV vaccination [25]. Our recent studies have shown that polymorphic variants of *IL*-*18* individually or jointly with polymorphic variants of *IL*-*12A* or *IL*-*12B* are associated with the development of anti-HBs in HD patients [26, 27]. It could not be distinguished from these studies [26, 27], whether there is any difference in the association between anti-HBs development and the examined polymorphic variants when anti-HBs are generated in response to HBV transmission after HBV clearance or when the protective immune humoral response is triggered by the vaccine selectively containing the S protein of HBsAg. This task seems to be especially meaningful in light of an earlier study showing that some inbred strains of mice that are unresponsive to protein S of HBsAg do produce anti-HBs when immunized with a larger surface viral protein containing S HBsAg and pre-S(1) [28]. Recombinant DNA hepatitis B vaccine containing HBsAg particles harbouring all three viral envelope polypeptides, the major S protein and the minor Pre-S2 and Pre-S1, was shown to be more powerful in the development of anti-HBs than did standard recombinant vaccines containing only S the protein [29].

The aim of our study was to perform a separate analysis of hepatitis B vaccinated and HBV infected HD patients in relation to the polymorphic variants of *IL12A* G>A (rs568408) and *IL12B* A>C (rs3212227) and to assess whether in HD patients polymorphic variants of *IL*-*12* are equally associated with the development of anti-HBs in the event of HBV vaccination or HBV infection.

## Materials and methods

### Patients and controls

Studies were carried out in 839 HD patients treated in 22 dialysis centers located in the Wielkopolska region of Poland. Metrical age and renal replacement therapy (RRT) vintage that are shown in the Results section are both in regards concern to the date that blood samples were collected for genotyping. HBV seromarkers (HBsAg, anti-HBc, anti-HBs) were determined in each patient at HD commencement. HBsAg determinations were repeated on a mandatory basis every 6 months, total anti-HBc voluntarily every 8–12 months. All patients were vaccinated against HBV with recombinant DNA yeast-derived vaccines, composed of the S protein of HBsAg (Engerix B, GlaxoSmithKline Biologicals, Belgium; Hepavax–Gene, BIOMED SA, Poland; Euvax B, LG Chemical, South Korea) according to the rules established for HD patients [1]. An anti-HBs titre was checked after 4–8 weeks from the last vaccine dose. When an anti-HBs titre remained below 10 IU/L, assumed to be non-protective in vaccinated patients [30], vaccination was repeated. The level of anti-HBs wanes over the time after vaccination, so a blood test for anti-HBs was repeated in all HD patients on a mandatory basis every 6 months to determine if vaccine booster doses were required.

### Patients enrolled to the study had to fulfill the following criteria:

- Treatment with HD due to end-stage renal disease,
- No signs and symptoms of acute infection with blood-borne viruses within 6 months before enrollment,
- Determined panel of HBV seromarkers sufficient for a classification of a patient to:
  - HBV vaccinated group (I) without (subgroup Ia) or with (subgroup Ib) developed anti-HBs,
  - HBV infected group (II) without (subgroup IIa) or with (subgroup IIb) developed anti-HBs,
- From patients who disclosed a genetic relationship only one person could participate in the study,
- Provided written consent to participate in the study.

Criteria of classification of HD patients to the aforementioned groups are presented in Table 1. Only patients with no history of acute hepatitis B and showing HBsAg and anti-HBc negative in all tests were included into group I. Therefore, there was no documentation that anti-HBs could be developed due to HBV transmission prior to immunization. Patients that were never vaccinated for hepatitis B that consistently maintained anti-HBc positivity, also showing isolated anti-HBs positivity, were considered to have been infected with HBV in the past (group II).

**Table 1:** Criteria of classification of hemodialysis patients

Parameter	Group I		Group II	
	Subgroup Ia	Subgroup Ib	Subgroup IIa	Subgroup IIb
History of acute hepatitis B	No		Yes or no	
HBsAg	Negative in all available results		Negative or positive	
Anti-HBc	Negative in all available results		Positive	
			in a patient with documented HBsAg currently or in the past,	
			in a never hepatitis B vaccinated patient with an anti-HBs titre >10 IU/L, confirmed by two independent laboratories using different methods of anti-HBc determination and maintained in consecutive determinations in a patient without other HBV seromarkers	
Hepatitis B vaccination	Two full vaccination series (4 doubled doses of 20 μg each given at 0–1–2–6 months) or equivalent vaccine summarized dose (160 μg) with anti-HBs titre ≤ 10 IU/L after 4–8 weeks from the last vaccine dose	At least one vaccination series	No hepatitis B vaccination	
Anti-HBs	Negative (≤10 IU/L) in all available results	Positive (>10 IU/L) in at least one confirmed result	Negative (≤10 IU/L) in all available results	Positive (>10 IU/L) in at least one confirmed result

*anti-HBc* antibodies to core antigen of hepatitis B virus, *anti-HBs* antibodies to surface antigen of hepatitis B virus, *HBsAg* surface antigen of hepatitis B virus

All of the available results for HBV seromarkers of each patient were analyzed. If a patient had an anti-HBs titre >10 IU/L in the past and further had a decline in an anti-HBs titre <10 IU/L, s/he was considered to be constitutionally able to develop anti-HBs and was included into group Ib or IIb.

Hepatitis B vaccinated or HBV infected HD patients, being also infected with hepatitis C virus (HCV), were included into the study when they were established as HBsAg responders or not responders before HCV transmission.

Group I included 602 patients (subgroup Ia-199, subgroup Ib-403), group II-237 patients (subgroup IIa-55, subgroup IIb-182).

Registered blood donors, qualified for blood donation according to the criteria of Polish Ministry of Health [31], served as controls for HD patients. All controls (*n* = 240) showed negative blood testing for HBsAg and HBV DNA as well as for seromarkers of infection with HCV. Unfortunately, the hepatitis B vaccination rate and an anti-HBs titre were not known in these healthy individuals.

Genotype analysis for rs568408 3′UTR G>A in *IL*-*12A* and rs3212227 3′ UTR A>C in *IL*-*12B* was performed in all patients and controls.

### Laboratory methods

HBV and HCV seromarkers, as well as serum activities of liver enzymes, were determined as previously described [27]. All HBV or HCV positive results were the subject of confirmation tests.

### *IL*-*12A* and *IL*-*12B* genotyping

DNA was isolated from peripheral leukocytes. The *IL*-*12A 3′UTR* G > A (rs568408) polymorphism was genotyped by high-resolution melting curve analysis (HRM); identification of the *IL*-*12B 3′UTR* A>C (rs3212227) polymorphic variants was carried out by polymerase chain reaction-restriction fragment length polymorphism (PCR–RFLP), as previously described [27]. For quality control of the tested polymorphisms, approximately 10 % of the randomly chosen samples were re-genotyped using commercial sequencing. We observed 99.9 % concordance between results of PCR-RFLP analysis and sequencing.

### Statistical methods

Descriptive statistics are presented as percentage for categorical variables, and median with range for continuous variables because asymmetry of distribution was shown in all but one (age in subgroup IIa) variable as tested by the Shapiro–Wilk test. The prevalence of variables was assessed by the Chi square test, the Chi square test with Yates correction, or the V square test, as appropriate. Continuous variables were compared using the Mann–Whitney U-test.

Hardy–Weinberg equilibrium was tested by the Chi square test with one degree of freedom (*P* < 0.01 for significance) to compare the observed genotype frequencies to the expected ones. Power analysis was conducted employing the Fisher exact test (http://biostat.mc.vanderbilt.edu/twiki/bin/view/Main/ PowerSampleSize).

The associations between genotypes and the anti-HBs phenotype were estimated by computing the odds ratio (OR) and its 95 % confidence interval (95 % CI). A frequency distribution of the examined genotypes was referred to that of the respective homozygous wild-type genotypes. Results of all associations were adjusted, if possible, for parameters which significantly differentiated the examined groups. Values of *P* < 0.05 were judged to be significant. All probabilities were two-tailed. The *P* value using the Bonferroni correction for multiple testing was calculated, if appropriate, and related to results of the initial statistical analysis. Logistic regression analysis was used to show variables associated with anti-HBs phenotype concomitantly with the examined polymorphic variant. To address the possibility of a gene–gene interaction effect between the analyzed SNPs, a nonparametric and genetic model-free multifactor dimensionality reduction (MDR) approach was used [32].

### Ethical issues

This study was approved by the Institutional Review Board of Poznań University of Medical Sciences, Poland.

## Results

### Results of HBV vaccinated HD patients (subgroups Ia and Ib)

The selected demographic, clinical and laboratory data of vaccinated HD patients are shown in Table 2. All patients were Caucasian.

**Table 2:** The selected demographic, clinical and laboratory data of vaccinated hemodialysis patients divided into subgroups

Parameter	Group I (*n* = 602)		*P* value for differences between Ia and Ib
	Subgroup Ia (*n* = 199)	Subgroup Ib (*n* = 403)	
Men, *n* (% of all)	103 (51.8)	228 (56.6)	0.296
Age, years	69.6 (23.8–92.5)	62.8 (18.6–91.7)	**<0.001**
RRT duration, years	1.0 (0.03–11.6)	2.8 (0.003–26.1)	**<0.001**
Diabetic nephropathy, *n* (% of all)	72 (36.2)	102 (25.3)	**0.007**
Chronic glomerulonephritis, n (% of all)	11 (5.5)	78 (19.4)	**<0.001**
Hypertensive nephropathy, *n* (% of all)	40 (20.1)	67 (16.6)	0.308
Chronic tubulointerstitial nephritis, *n* (% of all)	18 (9.0)	35 (8.7)	0.880
An anti-HBs titre >10 IU/L	–	403 (100 %)	NA
Positive anti-HCV, *n* (% of all)	11 (5.5)	37 (9.2)	0.150
Positive both anti-HCV and HCV RNA (*n*, % of all anti-HCV positive)	5 (45.5)	25 (67.6)	0.288
ALT (IU/L)	13 (0.6–126)	13 (2–209)	0.222
AST (IU/L)	14 (5–97)	15 (4–177)	0.309
GGT (IU/L)	30 (5–308)	25 (0–472)	0.590

Continuous variables are expressed as median and range. Significant results are indicated using bold font

*ALT* alanine aminotransferase, *anti-HBs* antibodies to surface antigen of hepatitis B virus, *anti-HCV* antibodies to hepatitis C virus, *AST* aspartate aminotransferase, *GGT* gamma-glutamyltranspeptidase, *HCV RNA* ribonucleic acid of hepatitis C virus, *NA* not applicable, *RRT* renal replacement therapy

Distribution of *IL*-*12A* and *IL*-*12B* polymorphic variants in subgroups Ia and Ib was in agreement with Hardy–Weinberg equilibrium. In group I, differences of genotype frequencies were adjusted, if possible, for age, RRT vintage, and diabetic nephropathy or chronic glomerulonephritis as causes of RRT.

In the logistic regressions that included gender, age, RRT vintage, kidney diseases, liver enzymes, anti-HCV, and polymorphic variants of *IL*-*12A*, there was a positive association of anti-HBs development in response to hepatitis B vaccination with concurrently RRT vintage (OR 1.3, 95 % CI 1.2–1.5, *P* < 0.001), chronic glomerulonephritis (OR 2.6, 95 % CI 1.2–5.4, *P* = 0.012), and GA *IL*-*12A* (OR 1.6, 95 % CI 1.0–2.5, *P* = 0.035); a negative association was shown with age (OR 0.98, 95 % CI 0.97–1.0, *P* = 0.018) (*P* < 0.001 for the significance of this model). Similar results were obtained if anti-HCV were replaced in the model by HCV RNA. If a frequency distribution of the GA rs568408 *IL*-*12A* variant was related to that of the homozygous wild-type genotype GG rs568408 *IL*-*12A*, patients bearing GA had a 1.6-fold higher chance to respond to the HBV vaccine than patients having the wild-type genotype (sample power 74 %). However, when the Bonferroni correction was applied this result was not significant (Table 3).

**Table 3:** *IL*-*12* polymorphisms in hemodialysis non-responders to hepatitis B vaccine (subgroup Ia) and hemodialysis responders to hepatitis B vaccine (subgroup Ib) with the development of antibodies to surface antigen of hepatitis B virus

Genotype	Subgroup Ia (*n* = 199) *n* (%)	Subgroup Ib (*n* = 403) *n* (%)	OR (95 % CI)	*P* value
*IL*-*12A*				
GG	152 (76.4)	275 (68.2)	Referent	
GA	40 (20.1)	115 (28.5)	1.6 (1.0–2.5)^a^	0.033^b^
AA	7 (3.5)	13 (3.2)	1.0 (0.6–1.7)^a^	0.984
GA/AA	47 (23.6)	128 (31.8)	1.5 (1.0–2.3)^a^	0.048^b^
AA	7 (3.5)	13 (3.2)	Referent	
GA/GG	192 (96.5)	390 (96.8)	1.2 (0.4–3.2)^a^	0.783
Allele G	344 (86.4)	665 (82.5)	Referent	
Allele A	54 (13.6)	141 (17.5)	1.4 (1.0–1.9)	0.096
*IL*-*12B*				
AA	121 (60.8)	231 (57.3)	Referent	
AC	74 (37.2)	160 (39.7)	1.0 (0.7–1.5)^a^	0.876
CC	4 (2.0)	12 (3.0)	1.1 (0.6–2.1)^a^	
AC/CC	78 (39.2)	172 (42.7)	1.3 (0.4–4.5)^a^	0.626
CC	4 (2.0)	12 (3.0)	Referent	
AC/AA	195 (98.0)	391 (97.0)	0.8 (0.2–2.6)^a^	0.660
AlleleA	319 (79.6)	622 (77.2)	Referent	
Allele C	82 (20.4)	184 (22.8)	1.2 (0.9–1.6)	0.387

^a^Odds ratio (OR) after adjustment for age, renal replacement therapy (RRT) vintage, and diabetic nephropathy or chronic glomerulonephritis as causes of RRT

^b^Non-significant after the Bonferroni correction for multiple comparisons (*P* > 0.017)

Statistical evidence for association of the *IL*-*12A* polymorphism with anti-HBs development in response to hepatitis B vaccination is summarized in Table 4.

**Table 4:** Statistical evidence for association of *IL*-*12A* polymorphism with anti-HBs development in response to hepatitis B vaccination

*IL*-*12A* polymorphism	Adjusted reference to homozygous wilde-type genotype		The logistic regression (OR, 95 % CI, *P*)
	Without the Bonferroni correction	With the Bonferroni correction	
	(OR, 95 % CI, *P*)	(OR, 95 % CI, *P*)	
GG	na	na	ns
GA	1.6, 1.0–2.5, 0.033	ns	1.6, 1.0–2.5, 0.035
AA	ns	ns	ns
GA/AA	1.5, 1.0–2.3, 0.048	ns	ns

*na* not applicable, *ns* non-significant

There was no association between the *IL*-*12B* polymorphism and anti-HBs phenotype (Table 3).

### Results of HBV infected HD patients (subgroups IIa and IIb)

The selected demographic, clinical and laboratory data of infected HD patients are shown in Table 5. All patients were Caucasian.

**Table 5:** The selected demographic, clinical and laboratory data of infected hemodialysis patients divided into subgroups IIa and IIb with comparison of results to those of vaccinated patients (subgroups Ia and Ib)

Parameter	Group II (*n* = 237)		*P* value for differences between IIa and IIb	*P* value for differences between Ia and IIa	*P* value for differences between Ib and IIb
	Subgroup IIa	Subgroup IIb			
	(*n* = 55)	(*n* = 182)			
Men, *n* (% of all)	35 (63.6)	101 (55.5)	0.351	0.128	0.857
Age, years	59.3 (19.3–87.7)	61.7 (18.9–90.4)	0.143	**<0.001**	0.691
RRT duration, years	3.6 (0.1–24.2)	2.5 (0.05–26.0)	0.086	**<0.001**	0.179
Diabetic nephropathy, *n* (% of all)	9 (16.4)	53 (29.1)	0.079	**0.005**	0.363
Chronic glomerulonephritis, *n* (% of all)	17 (30.9)	30 (16.5)	**0.032**	**<0.001**	0.423
Hypertensive nephropathy, *n* (% of all)	5 (9.1)	24 (13.2)	0.490	0.072	0.326
Chronic tubulointerstitial nephritis, *n* (% of all)	6 (10.9)	18 (9.9)	0.802	0.613	0.643
History of acute hepatitis B, *n* (% of all)	5 (9.1)	10 (5.5)	0.568	NA	NA
Positive HBsAg, *n* (% of all)	20^a^ (36.4)	4^b^ (2.2)	**<0.001**	NA	NA
Positive HBV DNA, *n* (% of all)	18 (32.7)	4 (2.2)	**<0.001**	NA	NA
Positive anti-HBc, n (% of all)	55 (100 %)	182 (100 %)	NA	NA	NA
Isolated positive anti-HBc, *n* (% of all anti-HBc positive)	35 (63.6)	–	NA	NA	NA
An anti-HBs titre >10 IU/L	–	182 (100 %)	NA	NA	1.000
Positive anti-HCV, *n* (% of all)	13 (23.6)	37 (20.3)	0.577	**<0.001**	**<0.001**
Positive HCV RNA *n* (% of all anti-HCV positive)	8 (61.5)	24 (64.9)	1.000	0.682	1.000
ALT (IU/L)	18 (4–53)	14 (0–195)	0.295	**0.011**	0.372
AST (IU/L)	16 (8–81)	16 (1–152)	0.224	**0.024**	0.389
GGT (IU/L)	23 (–7284)	25 (0–692)	0.762	0.915	0.958

Continuous variables are expressed as median and range. Significant results are indicated using bold font

*ALT* alanine aminotransferase, *anti-HBc* antibodies to core antigen of hepatitis B virus, *anti-HBs* antibodies to surface antigen of hepatitis B virus, *anti-HCV* antibodies to hepatitis C virus, *AST* aspartate aminotransferase, *GGT* gamma-glutamyltranspeptidase, *HBV* hepatitis B virus, *HBsAg* surface antigen of hepatitis B virus, *HBV DNA* deoxyribonucleic acid of hepatitis B virus, *HCV RNA* ribonucleic acid of hepatitis C virus, *NA* not applicable, *RRT* renal replacement therapy

Distribution of *IL*-*12A* and *IL*-*12B* polymorphic variants did not show deviation from Hardy–Weinberg equilibrium with exception of subgroup IIa in respect to *IL*-*12B* (*P* = 0.0002). Subgroup IIa also differed in *IL*-*12B* genotype frequencies from controls (*P* = 0.016), which showed the distribution of the *IL*-*12B* genotype in concordance with Hardy–Weinberg equilibrium and with a previously described control Caucasian population [22].

There was no association between the *IL*-*12A* polymorphism and anti-HBs phenotype (Table 6).

**Table 6:** *IL*-*12* polymorphisms in hemodialysis non-responders to hepatitis B virus (HBV) transmission (subgroup IIa) and hemodialysis responders to HBV transmission (subgroup IIb) with the development of antibodies to surface antigen of HBV

Genotype	Subgroup IIa (*n* = 55) *n* (%)	Subgroup IIb (*n* = 182) *n* (%)	OR (95 % CI)	*P* value
*IL*-*12A*				
GG	34 (61.8)	130 (71.4)	Referent	
GA	17 (30.9)	48 (26.4)	1.0 (0.4–2.2)^a^	0.954
AA	4 (7.3)	4 (2.2)	0.6 (0.2–1.3)^a^	0.182
GA/AA	21 (38.2)	52 (28.6)	0.8 (0.4–1.8)^a^	0.660
AA	4 (7.3)	4 (2.2)	Referent	
GA/GG	51 (92.7)	178 (97.3)	3.1 (0.6–16.4)^a^	0.177
Allele G	85 (77.3)	308 (84.6)	Referent	
Allele A	25 (22.7)	56 (15.4)	0.62 (0.4–1.0)	0.083
*IL*-*12B*				
AA	39 (70.9)	107 (58.8)	Referent	
AC	9 (16.4)	71 (39.0)	5.7 (1.9–17.2)^a^	0.002^b^
CC	7 (12.7)	4 (2.2)	0.2 (0.06–0.8)	0.015^b^
AC/CC	16 (29.1)	75 (41.2)	2.7 (1.1–6.2)^a^	0.022^c^
CC	7 (12.7)	4 (2.2)	Referent	
AC/AA	48 (87.3)	178 (97.8)	7.0 (1.7–28.7)^a^	0.006
Allele A	87 (79.1)	285 (78.3)	Referent	
Allele C	23 (20.9)	79 (21.7)	1.0 (0.6–1.8)	0.896

^a^ Odds ratio (OR) after adjustment for HBV surface antigen and chronic glomerulonephritis as a cause of renal replacement therapy

^b^Significant after the Bonferroni correction for multiple comparisons (*P* < 0.017)

^c^Non-significant after the Bonferroni correction for multiple comparisons (*P* > 0.017)

In the best regression models that included gender, age, RRT vintage, kidney diseases, liver enzymes, HBsAg, anti-HCV, history of hepatitis B, and polymorphic variants of *IL*-*12B* (*P* < 0.001 for a significance of each model), there was a positive association of anti-HBs development in response to hepatitis B infection with the AC polymorphic variant of *IL*-*12B* (OR 8.0, 95 % CI 2.6–24.9, *P* < 0.001); negative associations were shown with HBsAg positivity (OR 0.02, 95 % CI 0.003–0.07, *P* < 0.001) and the CC polymorphic variant of *IL*-*12B* (OR 0.1, 95 % CI 0.03–0.06, *P* = 0.011). Similar results were obtained if anti-HCV were replaced in the model by HCV RNA, and HBsAg by HBV DNA.

In group II, results were adjusted, if possible, for HBsAg and chronic glomerulonephritis as a cause of RRT. If a frequency distribution of the examined polymorphisms was related to that of the homozygous wild-type genotype and the Bonferroni correction was applied, AC rs3212227 *IL*-*12B* was positively associated with anti-HBs development (sample power 99.4 %), whereas CC rs3212227 *IL*-*12B* was predictive for negative anti-HBs phenotype (sample power 69.5 %, Table 6).

Selected dichotomized and combined effects of the *IL*-*12* polymorphisms are presented in Tables 7 and 8. Compared to any other genotypes, GG rs568408 *IL*-*12A* and AC rs3212227 *IL*-*12B* were associated with positive anti-HBs phenotype (sample power 90.8 %), whereas both GG rs568408 *IL*-*12A* and CC rs3212227 *IL*-*12B* (sample power 74.2 %) or both AA rs568408 *IL*-*12A* and AA rs3212227 *IL*-*12B* (sample power 66.0 %) were related to the negative anti-HBs phenotype (Table 7). When pairs of genotypes were referred to the other selected genotype pair, patients bearing both GG rs568408 *IL*-*12A* and CC rs3212227 *IL*-*12B* had a near-17-times lower chance to develop anti-HBs compared to patients having both GG rs568408 *IL*-*12A* and AC rs3212227 *IL*-*12B* (sample power 91.9 %, Table 8).

**Table 7:** Selected dichotomized effects of the *IL*-*12A* rs568408 and *IL*-*12B* rs3212227 polymorphisms in infected hemodialysis patients

Genotypes	Group IIa (*n* = 55), *n* (%)	Group IIb (*n* = 182), *n* (%)	OR (95 % CI)	*P* value
All other genotypes	48 (87.3)	129 (70.9)	Referent	
rs568408 GG and rs3212227 AC	7 (12.7)	53 (29.1)	4.0 (1.3–11.9)^a^	0.012
All other genotypes	53 (96.4)	181 (99.5)	Referent	
rs568408 GG and rs3212227 CC	2 (3.6)	1 (0.5)	0.2 (0.03–0.8)^a^	0.025
All other genotypes	53 (96.4)	181 (99.5)	Referent	
rs568408 AA and rs3212227 AA	2 (3.6)	1 (0.5)	0.09 (0.008–1.0)^a^	0.049

^a^Odds ratio (OR) after adjustment for HBV surface antigen and chronic glomerulonephritis as a cause of renal replacement therapy

**Table 8:** Selected combined effects of the *IL*-*12A* rs568408 and *IL*-*12B* rs3212227 polymorphisms in infected hemodialysis patients

Genotypes	Group IIa (*n* = 55) Frequency (%)	Group IIb (*n* = 182) Frequency (%)	OR (95 % CI)	*P* value
rs568408 GG and rs3212227 AA	24 (43.6)	74 (40.7)	Referent	
rs568408 GG and rs3212227 AC	7 (12.7)	53 (29.1)	4.6 (1.3–16.5)^a^	0.019^c^
rs568408 GG and rs3212227 AC	7 (12.7)	53 (29.1)	Referent	
rs568408 GG and rs3212227 CC	4 (7.3)	3 (1.6)	0.06 (0.008–0.4)^a^	0.005^b^
rs568408 AA and rs3212227 AA	2 (3.6)	1 (0.5)	0.5 (0.3–0.8)^a^	0.009^c^

^a^Odds ratio (OR) after adjustment for HBV surface antigen and chronic glomerulonephritis as a cause of renal replacement therapy

^b^Significant after the Bonferroni correction for multiple comparisons (*P* < 0.006)

^c^Non-significant after the Bonferroni correction for multiple comparisons (*P* > 0.006)

The MDR analysis revealed a borderline statistical significance between subgroups IIa and IIb. In this case, the testing balanced accuracy for the analysed 2-locus model was 0.582, cross validation consistency 100 % and *P* value derived from the 1,000-fold permutation test was 0.082.

Statistical evidence for an association of the *IL*-*12B* polymorphism with anti-HBs development in response to hepatitis B virus infection is summarized in Table 9.

**Table 9:** Statistical evidence for association of *IL*-*12B* polymorphism with anti-HBs development in response to hepatitis B virus infection

*IL*-*12B* polymorphism	Adjusted reference to homozygous wilde-type genotype		The logistic regression (OR, 95 % CI, *P*)
	Without the Bonferroni correction (OR, 95 % CI, *P*)	With the Bonferroni correction (OR, 95 % CI, *P*)	
AA	na	na	0.3, 0.1–0.7, 0.007
AC	5.7, 1.9–17.2, 0.002	5.7, 1.9–17.2, 0.006	8.0, 2.6–24.9, <0.001
CC	0.2, 0.06–0.8, 0.015	0.2, 0.06–0.8, 0.045	0.1, 0.03–0.6, 0.011
AC/CC	2.7, 1.1–6.2, 0.022	ns	3.3, 1.4–7.8, 0.007

*na* not applicable, *ns* non-significant

### HCV infection in HD patients

HD patients with an active HCV infection were equally distributed among individuals that developed anti-HBs and those that did not develop anti-HBs. This was the case for both vaccinated (Table 2) and infected (Table 5) HD patients. The logistic regression analysis did not reveal a significant predictive value of anti-HCV or HCV RNA for anti-HBs development in hepatitis B vaccinated or infected patients.

### Comparison of results of HD patients that did not develop anti-HBs despite HBV vaccination or infection (subgroups Ia and IIa)

Comparison of the *IL*-*12A* and *IL*-*12B* polymorphic variants in subgroups Ia and IIa revealed that a higher frequency of allele G rs568408 *IL*-*12A* remained associated with non-responsiveness to hepatitis B vaccination (OR 0.5, 95 % CI 0.3–0.9, *P* = 0.025), whereas the negative anti-HBs phenotype after HBV transmission remained associated with a higher frequency of CC rs3212227 *IL*-*12B* (adjusted OR 4.6, 95 % CI 1.0–21.1, *P* = 0.047) and lower frequencies of AC (adjusted OR 0.4, 95 % CI 0.2–1.0, *P* = 0.039) and AC/AA (adjusted OR 0.2, 95 % CI 0.05–1.0, *P* = 0.047) compared to any other examined genotypes of *IL*-*12B*.

### Comparison of results of HD patients that developed anti-HBs as a result of HBV vaccination or infection (subgroups Ib and IIb)

Comparison of subgroups Ib and IIb in respect to the *IL*-*12A* and *IL*-*12B* polymorphic variants showed that patients who developed anti-HBs in response to hepatitis B vaccination showed higher frequencies of GA rs568408 *IL*-*12A* (adjusted OR 2.4, 95 % CI 1.2–4.9, *P* = 0.015) and GA/AA (adjusted OR 2.5, 95 % CI 1.3–5.0, *P* = 0.007), and a lower frequency of GG rs568408 *IL*-*12A* (adjusted OR 0.4, 95 % CI 0.2–0.8, *P* = 0.007) compared to any other examined genotypes of *IL*-*12A*.

## Discussion

Gene polymorphisms of many cytokines have been established independently as being associated with a response to inoculation with the hepatitis B vaccine [25, 33–36] or with HBV clearance after natural infection [24, 37–42]. Our study suggests that cytokine gene polymorphisms associated with a positive anti-HBs phenotype may be different in the case of vaccination than those shown in the case of HBV infection.

### *IL*-*12* polymorphism in vaccinated HD patients

The presence of deficient Th1-like cells is mentioned among the causes of non-responsiveness to hepatitis B vaccination [43–45]. *IL*-*12* is a Th1 response agonist. Peripheral blood mononuclear cells from high responders to HBV vaccines show an elevated production of *IL*-*2*, *IL*-*12*, and interferon (IFN)-gamma [45, 46]. In dialysis patients, however, the deficit of IFN-gamma has been noted, despite increased levels of serum *IL*-*12* [47] or plasma free IL-18 [48]. These findings suggest the importance of proper genetic regulation of cytokine production and function in uremic patients.

In this study, multiple statistical analyses, with included a calculation of OR with 95 % CI, adjustment for possible confounding variables and logistic regression analysis, indicated that GA rs568408 *IL*-*12A* may be the probable polymorphic variant individually associated with anti-HBs development in vaccinated HD patients. An application of the Bonferroni correction for multiple testing showed, however, that the association of GA *IL*-*12A* with the anti-HBs phenotype is too weak to be significant after correction. Although the Bonferroni correction is widely used, there is a criticism to this method [49] and not all authors are willing to use it in genetic studies [25]. Further studies with the inclusion of more patients may reach a statistical significance with the Bonferroni correction and may elucidate the significance of the association between rs568408 *IL*-*12A* and a response to the hepatitis B vaccine. To our knowledge, we took the first step in this analysis.

In 2004, the *IL*-*12B* promoter S allele was associated with the non-responsiveness to hepatitis B vaccination, whereas the promoter L allele, 3′ UTR A and 3′ UTR C were not associated with the responder/non-responder phenotype. The heterozygosity of the *IL*-*12B* promoter was a significant contributor to the non-responder phenotype [25]. In our study *IL*-*12B* rs3212227 3′UTR A>C was not individually associated with the responder/non-responder phenotype after hepatitis B vaccination.

It is worth mentioning that HD patients frequently exhibit a deteriorated clinical status due to uremic, dialysis-related, and incidental complications. Hepatitis B vaccination is being performed (and repeated as needed) in the most optimal clinical status of each individual HD patient. Moreover, if the clinical status of a hepatitis B vaccine non-responder improves significantly, a booster dose is given by many clinicians, despite a previous full vaccination series in these patients. This practice may explain why the positive anti-HBs phenotype is associated with longer RRT vintage [50, this study]. Additionally, hepatitis B vaccine responders were younger and the prevalence of diabetic nephropathy was less frequent in this group, whereas chronic glomerulonephritis as a cause of RRT was more frequent. Patients dialyzed due to primary renal disease are usually in much better general condition than patients suffering from multi-organ diseases, such as diabetes mellitus. Older age, shorter RRT vintage, and diabetes mellitus are well-known risk factors associated with non-responsiveness to hepatitis B vaccination [50–52]. To decrease the impact of confounding variables on the results of genotype distribution analysis, OR were adjusted for age, RRT vintage, and the main causes of RRT. There were no differences in gender distribution between responders and non-responders, as shown in previous studies [53, 54]. Infection with HCV was also documented as a probable cause of hepatitis B vaccination failure [55], but in this study we did not observe a significant influence of HCV infection on anti-HBs development, because patients were established as responders or non-responders prior to HCV infection. Furthermore, it has been shown that HCV patients secrete normal amounts of *IL*-*12* [46].

Theoretically, genetic investigations could help in the development of improved hepatitis B vaccines that may eventually reduce the proportion of vaccine failures [56]. The use of exogenous *IL*-*12* as an adjuvant to augment anti-HBs development in response to hepatitis B vaccines has previously been discussed [46, 57]. Our data do not provide clear confirmatory evidence that such an action at the genetic level could be evidently helpful in the case of *IL*-*12* polymorphic variants.

### *IL*-*12* polymorphism in infected HD patients

An increase of serum *IL*-*12* level was associated with more effective HBV DNA clearance in patients with chronic hepatitis B [58], but no association with HBV persistence and *IL*-*12A* exon 7 +6400 C>T, +6624 G>A, 3′UTR +7003 T>C SNPs and haplotype of *IL*-*12A* +6400/+6624/+7003 was shown in the study by Park et al. [24]. In our study, the *IL*-*12A* rs568408 3′UTR G>A was not individually associated with anti-HBs development after HBV transmission. Therefore, the *IL*-*12A* polymorphism seems not to be involved in HBV elimination and development of protective anti-HBs. Different results were shown in the case of the *IL*-*12B* rs3212227 3′UTR A>C: the AC genotype of *IL*-*12B* was associated with a positive anti-HBs phenotype after HBV infection in HD patients. Individuals heterozygous in *Taq*I RFLP at position 1188 in the 3′URT of the *IL*-*12B* p40 gene (rs3212227) showed intermediate secretion of *IL*-*12* p70 after stimulation of monocytes with *Staphylococcus aureus* strain Cowan and IFN-gamma compared to homozygous individuals [23]. Dichotomized and combined effects of *IL*-*12A* and *IL*-*12B* genotypes confirm that *IL*-*12B* polymorphic variants have priority in the determination of anti-HBs phenotype in HBV infected HD patients.

The deviation of rs3212227 *IL*-*12B* polymorphic variants from Hardy–Weinberg equilibrium in HBV infected HD patients of subgroup IIa needs to be discussed. The *IL*-*12B* polymorphism was consistent with Hardy–Weinberg equilibrium in subgroup IIb and controls. This may suggest that significant differences in the characteristics of subgroups IIa and IIb could be involved in the observed discrepancy. As expected from the natural course of HBV infection, anti-HBc positive patients that did not develop anti-HBs are more frequently HBsAg/HBV DNA positive compared to patients with developed protective anti-HBs. The coexistence of positive HBsAg and anti-HBs was described for 9–21 % of chronic HBV carriers [59, 60]. In our study, this coexistence occurred in about 17 %. Subgroup IIa included also a higher percentage of patients with chronic glomerulonephritis compared to subgroup IIb. As already mentioned, the former group also showed a higher prevalence of persistent HBV infection as indicated by the positive HBV DNA tests. Glomerulonephritis is an important extrahepatic manifestation of chronic HBV infection [61], and this etiology was probable also in the examined HBV DNA positive patients, especially considering that in the Wielkopolska region of Poland the majority (82.1 %) of HBsAg positive HD patients underwent HBV infection prior to dialysis commencement [62]. Therefore, these factors (HBsAg/HBV DNA positivity, chronic glomerulonephritis prevalence) are closely related to the type of manifestation of HBV infection, and the heterogeneity of both subgroups in this respect is a consequence of patients’ categorization as anti-HBs positive or negative. Interestingly, significant gender differences did not occur between HBV infected patients with either the positive or negative anti-HBs phenotype. In most human populations, women are more likely than men to produce anti-HBs in response to HBV vaccination [63]. HBV infected women receiving dialysis treatment also developed anti-HBs more frequently than men [64]. Isolated anti-HBc positivity seems not to be gender-dependent [65, 66]. HBV infected HD patients without developed anti-HBs (subgroup IIa) had predominantly isolated anti-HBc positivity (63.6 %), therefore the higher prevalence of men (63.6 %) in this group did not reach significance in comparison with men that developed anti-HBs (55.5 %, subgroup IIb).

Additionally, subgroup IIa was small, because the selection criteria for inclusion into it were very specific. Until the year 2009 in Poland, when the results of anti-HBc testing were predominantly not known, HBsAg negative/anti-HBc positive patients were hepatitis B vaccinated using a standard procedure. Because patients with isolated anti-HBc positivity may develop anti-HBs in some cases in response to vaccination [67, 68], we have qualified to group II only patients who were not vaccinated to be sure that the immune response is affected only by natural infection and not vaccination. Moreover, to find individuals who are not vaccinated for HBV will become more difficult each year in Poland, because all newborns are vaccinated on a mandatory basis. On the other hand, sample power frequently exceeded 80 % in comparisons between subgroups IIa and IIb in respect to the rs3212227 *IL*-*12B* polymorphic variants. All of these facts may suggest that the lack of agreement with Hardy–Weinberg equilibrium has to be considered as a true difference between the examined groups.

## Conclusions

1. *IL*-*12A* and *IL*-*12B* polymorphic variants are associated in Caucasian HD patients with the anti-HBs phenotype.
2. The GA rs568408 *IL*-*12A* variant seems to be predictive for the development of protective immunization in response to hepatitis B vaccination in HD patients.
3. The AC rs3212227 *IL*-*12B* polymorphism is a predictor of favorable outcome after HBV infection in HD patients. HD patients that do not develop anti-HBs after HBV infection (HBsAg carriers or individuals with isolated anti-HBc serum profile) more frequently bear the CC rs3212227 *IL*-*12B* (12.7 % vs 2.2 %) polymorphic variant than patients with HBV-induced anti-HBs development.

## Abbreviations

ALT: Alanine aminotransferase
anti-HBc: Antibodies to core antigen of hepatitis B virus
anti-HBs: Antibodies to surface antigen of hepatitis B virus
anti-HCV: Antibodies to hepatitis C virus
AST: Aspartate aminotransferase
CI: Confidence interval
DNA: Deoxyribonucleic acid
GGT: Gamma-glutamyltranspeptidase
HBV: Hepatitis B virus
HBsAg: Surface antigen of hepatitis B virus
HCV: Hepatitis C virus
HD: Hemodialysis
IL: Interleukin
IFN: Interferon
MDR: Multifactor dimensionality reduction
MEIA: Microparticle enzyme immunoassay
NA: Not applicable
OR: Odds ratio
PCR–RFLP: Polymerase chain reaction–restriction fragment length polymorphism
RNA: Ribonucleic acid
RRT: Renal replacement therapy
SNP: Single nucleotide polymorphism
UTR: Untranslated region
